# Beyond age: frailty, shared decision-making and recovery trajectories in very old critically Ill patients

**DOI:** 10.1007/s41999-026-01571-2

**Published:** 2026-08-27

**Authors:** Jean-Pierre Michel, Athanase Benetos

**Affiliations:** 1 Centre Médical Univeristaire, University of Geneva, Geneva, Switzerland; 2 Geriatric Department, University of Lorraine, CHRU of Nancy, Nancy, France

**Keywords:** Geriatrics, Reanimation, Care trajectory, Frailty, End of Life, Ethics

Population aging is transforming intensive care medicine worldwide. Adults aged 80 years and older now represent a rapidly growing proportion of admissions to intensive care units (ICUs), challenging traditional models of critical care that were largely developed in younger populations. At the same time, advances in critical care have improved short-term survival, raising new questions regarding the appropriateness, goals, and outcomes of intensive care in very old patients.

Historically, chronological age has often influenced decisions regarding ICU admission, escalation of treatment, and allocation of healthcare resources. However, an increasing body of evidence demonstrates that age alone is a poor predictor of outcome. Older adults constitute a remarkably heterogeneous population. Individuals of the same chronological age may differ substantially in physiological reserve, functional capacity, cognitive status, resilience, social support, and ability to recover after acute illness. Consequently, chronological age should never be considered a sufficient criterion for either ICU admission or exclusion.

Among the factors influencing outcomes in very old critically ill patients, frailty has emerged as one of the most relevant and clinically useful concepts. Frailty reflects a state of increased vulnerability resulting from the cumulative decline of multiple physiological systems and is consistently associated with mortality, functional deterioration, institutionalization, and reduced quality of life. Importantly, frailty provides a more meaningful estimate of biological reserve than chronological age alone. Nevertheless, frailty should not be viewed simply as a prognostic score. Rather, it should serve as the entry point to a broader, person-centered assessment that integrates functional independence, cognition, multimorbidity, nutritional status, medication burden, social circumstances, and patient priorities.

The growing recognition of frailty has profound implications for decision-making in critical care. This avoids, on the one hand, withholding more intensive therapeutic interventions from robust elderly patients due to fatalism or an excessive fear of complications and, on the other hand, prevents the use of aggressive and futile treatments in the frailest patients by identifying situations where the treatment's benefit-risk ratio is unfavorable. Thus, frailty assessment aims to personalize therapeutic strategies based on the patient's functional status and reserves, rather than their chronological age. In addition, this approach indicates that the key clinical question is no longer whether a patient is sufficiently old to justify treatment limitation, but whether intensive care is likely to contribute to outcomes that the individual patient would consider acceptable and meaningful. This shift requires clinicians to move beyond a purely disease-centered approach toward a more holistic evaluation of the person and their life trajectory.

Shared decision-making represents an essential component of this evolution. Decisions regarding ICU admission, continuation of life-sustaining therapies, and treatment limitation frequently occur under conditions of uncertainty. In such situations, integrating medical expertise with patient values and preferences becomes paramount. Whenever possible, critically ill older adults should participate actively in decisions regarding their care. Advance care planning, advance directives, and previously expressed wishes should be sought systematically and incorporated into clinical deliberations. When decision-making capacity is impaired, family members and surrogate decision-makers play a critical role in helping healthcare professionals understand the patient's values, goals, and acceptable trade-offs between survival, function, and quality of life. The concept of shared decision-making extends beyond the moment of ICU admission. It should accompany the patient throughout the continuum of care, including discussions regarding treatment escalation, limitation of life-sustaining interventions, rehabilitation goals, and discharge planning. Transparent, compassionate, and continuous communication is therefore not an adjunct to critical care but a core component of high-quality practice.

Equally important is the recognition that care of very old critically ill patients cannot be delivered by intensivists alone. The complexity of this population requires close collaboration between intensivists and geriatricians at every stage of the care pathway. Geriatric expertise contributes essential insights into frailty, multimorbidity, cognitive impairment, functional reserve, polypharmacy, and long-term outcomes, while intensivists provide expertise in acute organ support and management of life-threatening conditions. Together, these disciplines can develop a more balanced and individualized approach to care. This partnership should ideally begin before ICU admission whenever possible, continue throughout the acute phase of critical illness, and remain active during recovery and follow-up. Such collaboration is particularly important because many outcomes that matter most to older adults extend far beyond hospital survival. Functional independence, cognitive preservation, symptom burden, social participation, and quality of life may ultimately be more relevant than mortality alone.

The same principle applies to multidisciplinary care. Nurses, physiotherapists, occupational therapists, pharmacists, nutrition specialists, psychologists, social workers, rehabilitation professionals, and primary care physicians all contribute essential expertise. Their coordinated involvement is critical for preventing complications, preserving mobility and cognition, supporting families, and facilitating recovery. Increasing attention is also being directed toward the prevention and management of post-intensive care syndrome, which may have profound consequences for physical, cognitive, and psychological function in older survivors.

Recent work has highlighted the importance of adopting a trajectory-based perspective when evaluating outcomes in very old ICU patients. Traditional measures such as ICU mortality, hospital mortality, or length of stay provide only a partial understanding of patient outcomes. Recovery after critical illness often extends over months or years and follows highly heterogeneous trajectories influenced by baseline vulnerability, severity of illness, treatment exposures, rehabilitation opportunities, and social determinants of health.

Viewing critical illness through the lens of recovery trajectories represents a conceptual shift with important clinical implications. Opportunities to influence outcomes exist before, during, and after ICU admission. Prehabilitation strategies may improve physiological reserve before planned interventions. During critical illness, protective approaches aimed at preserving neurological and neuromuscular function may reduce long-term disability. After ICU discharge, structured rehabilitation, geriatric assessment, intermediate care units, acute geriatric wards, and community-based follow-up may all contribute to improved functional recovery and quality of life.

End-of-life care must also be considered an integral component of geriatric critical care. The increasing ability to sustain physiological functions does not eliminate the obligation to evaluate whether treatments remain beneficial. Avoiding non-beneficial interventions is both an ethical and clinical responsibility. Decisions to withhold or withdraw life-sustaining therapies should be grounded in careful assessment of prognosis, frailty, functional status, and patient preferences. Such decisions should always be accompanied by meticulous symptom control, palliative care when appropriate, and ongoing support for families and caregivers.

Taken together, these developments suggest that the field is moving toward a new paradigm of geriatric critical care. This paradigm is characterized by individualized assessment rather than age-based categorization, by frailty-informed rather than age-driven decision-making, by multidisciplinary and geriatric-integrated care, and by an emphasis on recovery trajectories rather than survival alone.

The goal is not merely to prolong life but to support outcomes that remain meaningful to older persons themselves. Success should therefore be measured not only in terms of mortality reduction but also in preservation of dignity, autonomy, function, cognition, social participation, and quality of life. The most relevant question is no longer whether a very old patient can survive intensive care. Rather, it is whether intensive care can help that individual achieve a recovery trajectory that remains compatible with his or her values, goals, and expectations.

In this sense, the future of critical care for very old patients lies not in age-based medicine, but in person-centered, frailty-informed, and trajectory-oriented care.

